# A fine resolution dataset of accessibility under different traffic conditions in European cities

**DOI:** 10.1038/s41597-020-00619-7

**Published:** 2020-08-25

**Authors:** Aris Christodoulou, Lewis Dijkstra, Panayotis Christidis, Paolo Bolsi, Hugo Poelman

**Affiliations:** 1 Joint Research Centre, European Commission, Seville, Spain; 2 DG Regional and Urban Policy, European Commission, Brussels, Belgium

**Keywords:** Geography, Interdisciplinary studies

## Abstract

Urban accessibility and congestion indicators allow us to benchmark cities. If these indicators are also available at a fine resolution, we can compare different neighbourhoods within a city. We present a dataset of different accessibility indicators for all urban areas with more than 250 thousand people in the EU27, the UK, Switzerland and Norway. Each city is analysed by means of a population grid of 500 m by 500 m and represented by a wider area covering both the densely populated urban centre and the commuting zone. To capture congestion, we measure accessibility for each grid cell at different times of the day that correspond to different traffic conditions using the detailed network and congestion information provided by TomTom.

## Abstract (summary)

**Table:** 

Measurement(s)	urban accessibility • congestion impact
Technology Type(s)	computational modeling technique
Factor Type(s)	longitude and latitude • urban area • transport performance • absolute accessibility • potential accessibility • location indicator
Sample Characteristic - Environment	city
Sample Characteristic - Location	Germany • Italy • French Republic • Poland • Sweden • Croatia • Kingdom of Spain • Kingdom of the Netherlands • Romania • Greece • Bulgaria • Kingdom of Denmark • Republic of Ireland • Belgium • Portuguese Republic • Austria • Hungary • Finland • Czech Republic • Island of Cyprus • Luxembourg • Slovenia • Malta • Lithuania • Slovak Republic • Estonia • Latvia • United Kingdom • Switzerland • Kingdom of Norway

Machine-accessible metadata file describing the reported data: 10.6084/m9.figshare.12687836

## Background & Summary

People have been moving to cities attracted by the agglomeration benefits associated with easy access to other people, jobs, opportunities, amenities etc. An indirect effect of the increasing population in cities is road congestion, resulting from more road vehicles sharing the fixed capacity of infrastructure. Increasing road capacity, however, does not necessarily reduce congestion in the medium-term as people adapt to the new situation by travelling longer distances and travelling more by car. More and longer car trips also increase greenhouse gas emissions and air pollution. Congestion strongly influences accessibility, but varies widely over time and across space. To capture this variation, the analysis of urban accessibility requires detailed spatial and temporal information on population, traffic conditions and transport infrastructure. We created a unique dataset of accessibility that covers all European cities with more than a quarter million inhabitants at fine resolution level considering different traffic conditions.

Accessibility has been in the epicentre of urban and regional transport policy-making. It provides a straightforward measure of the efficiency and effectiveness of transport infrastructure, but it can only be as informative as the data used to calculate it allow. The majority of relevant studies have been focusing on accessibility at regional level^1–3^ or measuring access to cities represented by a single point. Two noteworthy studies are Weiss *et al*.^4^ presenting a global map of travel time to cities followed up by the work of Nelson *et al*.^5^ that measures access to cities – more specifically to the boundary of cities, i.e. to the edge of a polygon and not to a point – considering size and modelling transport in a stylised way using friction surfaces. Both use the same city definition as this study. The first global map of travel times to major cities was developed by the Joint Research Centre of the European Commission and the World Bank in 2008^6^.

The collection and production of high-resolution data, and the increase of computing power have improved significantly the potential to calculate accessibility at fine resolution level^7^ taking into consideration the changes during a day in cities^8,9^.

Working at fine resolution not only improves the quality of the analysis, i.e. measuring accessibility in this case, but also enhances the analytical opportunities. For example, this data can identify the most or least accessible areas in the city, measure different aspects of accessibility and account for issues with strong spatial (and temporal) features, such as congestion. This is why we believe that the dataset presented here can be of wide interest and have several applications.

The dataset covers all Functional Urban Areas (FUA) – 310 in total – with more than 250 thousand people (2011 population^10^) in EU27, the UK, Switzerland and Norway. A FUA consist of a the city and its commuting zone^11^. Within these FUAs, we use population grid cells of 500 m by 500 m to calculate the indicators. Travel time from each grid cell to all others is calculated at different times of the day to capture the variation in traffic conditions. We rely on the full road network and link-level congestion information provided by TomTom.

The final dataset contains a set of accessibility indicators for each populated grid cell in the 310 European FUAs. The following four accessibility indicators are included: potential accessibility, location indicator, absolute accessibility and transport performance.

Calculating these indicators for all inhabited grid cells of all major European FUAs using the full road network and at different times of the day is very computationally demanding. For the routing calculations to find the shortest (quickest) path from each grid cell to all others, we used Python scripts and relevant libraries, while for the pre-processing we used ArcGIS. The approach was first tested for Seville, Krakow and Brussels^12^ and then applied to estimate accessibility for the remaining 307 FUAs.

We validated the travel time estimates against Google maps journey times, for a sample of origin-destination pairs drawn from all the cities.

The dataset can have several applications such as benchmarking European cities according to congestion and accessibility, analysing spatial patterns of congestion in relationship to urban forms, indicating with precision the areas in a city most affected by congestion etc. Combinations with other data – e.g. information regarding public transport or services – creates further opportunities for analysis.

## Methods

Accessibility indicators take into account the number of opportunities one can reach and how difficult it is to reach them. Opportunities represent the attractiveness of a location as a destination: for commuting trips, the appeal of a location could be the number of jobs, for shopping trips the number of shops, etc. In this paper, we use residential population as a proxy for the availability of opportunities, because we have access to detailed data based on official data. Population living outside the FUA is not taken into account. Day-time population would be a better proxy, but that is not yet available on a pan-European level using sufficiently detailed input data such as workplace based employment, students per school and/or mobile phone locations.

We calculate accessibility by car for each populated grid cell based on the driving time to all other populated grid cells in the FUA. Travel time is calculated at different times of a weekday – more specifically Tuesday – to take into account the variation of traffic over the course of a day and to capture the impact of congestion. The dataset includes two sets of results: the one refers to free flow conditions and the other to the morning peak of congestion.

The goal of this paper is to provide a comprehensive dataset of accessibility and congestion indicators for all major European cities using detailed road network and traffic data. Although a large share of daily trips in FUAs is made by public transport, active modes or a combination of different modes, it is beyond the scopes of this study to measure accessibility using other modes or considering different alternatives for door-to-door trips.

### Data used

The analysis is conducted at the level of FUAs that include both the city and its commuting zone^11^. Accessibility combines travel time between origin and destination zones with size of population, while trips between all zones are considered.

### Origin-destination zones within the cities

The FUA population is distributed to 500 m by 500 m grid cells. The data are based on the 1 km^2^ population grid for year 2011 provided by EUROSTAT^10^ which were the most recent grid population data available at the moment of the analysis. From the 1 km^2^ grid a 100 m by 100 m grid has been derived^13^. The population estimates from the 100 m grid have been summed to 500 m by 500 m grid cells. From the population grid covering all Europe only the cells included in the FUAs with more than a quarter million people have been selected. This is the case for 310 cities.

These cities vary considerably in terms of population and number of grid cells to which the population is distributed. For example, the FUA of Brussels has 2.5 million residents distributed to almost ten thousand grid cells, Krakow has less than 1.4 million people distributed to more than ten thousand grid cells and the population of the FUA of Madrid is more than 6.5 million distributed to six and a half thousand grid cells. The largest FUAs are those of London and Paris with approximately twenty thousand grid cells and 11 million population.

### Road network

Travel times between grid cells are calculated across the quickest routes on the full road network. The detailed representation of the road network by Multinet^14^ allows to model routing precisely with the help of variables such as the following: classification of roads according to importance (e.g. highway, major road etc.) or according to type (e.g. normal road, assistance lane, parking ramp etc.), state of the road (e.g. construction status), geo-locational characteristics, direction of the road link, length of the road link, speed based on classification and type of the road etc.

We calculate travel times at different hours of the day to capture the change of driving speed due to traffic conditions. TomTom^14^ provides data on relative speed corresponding to the percentage reduction of free flow speed due to traffic – free flow speed is measured in no-traffic conditions. Such data are provided for the road segments where there is a sufficient number of GPS measurements by vehicles (probes) to estimate daily speed variations and they are used to associate these links with one of 293 available speed profiles. Speed profiles correspond to a typical week of the year. They represent different patterns of variation of relative speed during a day and include 288 values of relative speed for one day, i.e. one value for every five minutes. Speed profiles and relative speed information are available for almost all highways and major roads in European cities while the coverage of the rest of the road classes varies from city to city.

### Data pre-processing

The road network data were prepared using Python and ArcGIS to compute travel times and accessibility for each city independently. The road network of each FUA, and the surrounding area required to ensure connectivity, was stored in a separate file. Hence, it has been possible to run calculations for many cities in parallel. For this purpose, in ArcMap we joined spatially the shapefile of the complete road network with the shapefile of FUAs in Europe. In order to ensure network connectivity, for the selection of the road network we extended the limits of each FUA to the rectangle embedding the FUA’s polygon. Finally, a separate file was created with the road network of each FUA.

Speed profiles at the link level capture the speed variation over the course of a day. Once we selected each FUA’s network, we created a separate file with the traffic information for the links of the FUA.

### Calculation of travel time

The calculation of driving time at this level of detail for many cities in parallel is very demanding computationally. We used the programming language Python and the NetworkX library for the development of network analysis algorithms. The road network is represented as a directed multigraph and every edge is associated with driving times corresponding to different traffic conditions. These driving times were used to calculate the shortest paths between origins and destinations.

We ran the calculations in a computer with 40 cores of 2.2 GHz and 128GB memory. For the majority of the cities considered it has been possible to have 40 instances running in parallel. Only during the processing of the bigger cities that required a lot of memory, the number of cores utilised had to be reduced.

The process to estimate shortest paths between populated grid cells is summarised in the following:

- Speed in traffic conditions is calculated by averaging relative speed over the selected time period, hourly in this case. Then, for each road link, driving times in free flow and congestion conditions are estimated.
- The full road network is loaded as a directed multigraph and the driving times calculated in the previous step are added as attributes of the links. They serve as the weights on which the shortest path estimation is based.
- The shortest paths from all origins to all destinations are calculated using the Dijkstra algorithm^15^ for different traffic (or no-traffic) conditions. Origins and destinations are the centroids of the grid cells and the actual shortest path calculated for each journey is the one between the closest nodes of the road network to the origin and destination respectively. Hence, travel times are calculated between all combinations of grid cells’ centroids in each FUA using the full road network.

To identify the peak congestion hour, we calculated travel times at different morning hours. The peak hour was the one with the lowest accessibility. For the vast majority of cities, the morning peak of congestion is between 08:00–09:00 am (local time). We used this time slot for all cities.

Now, we can calculate accessibility using different operationalisations to express the relationship with the estimated travel times and grid population.

### Accessibility indicators

We calculated four accessibility indicators that quantify different underlying aspects relevant to the spatial relationships within cities:

- Absolute accessibility: an absolute measure of opportunities reachable within a certain travel time
- Transport performance: a relative measure of opportunities controlling for the size of city
- Location indicator: a measure of a zone’s connectivity
- Potential accessibility: a measure of a zone’s access to all opportunities

The formulation of the indicators^16–19^ is the following:

Absolute accessibility measures accessible population within a certain travel time. Congestion has a negative impact on absolute accessibility by increasing travel time i.e. reducing the number of destinations reachable within the specified limit. The formula is as follows:

$$A{A}_{i\kappa }=\mathop{\sum }\limits_{j=1}^{n}{P}_{j}{\delta }_{ij}$$

where:

*P*_*j*_ the population of destination zone *j*;

${\delta }_{ij}$ a binary variable equal to 1 when travel time from zone *i* to zone *j* is smaller than the determined travel time limit *κ* (30 minutes in our case) and 0 otherwise, and

*n* the number of destination zones to be taken into account in the calculation (all in the specific case).

The 30 min time limit in absolute accessibility was used as the reference cut-off-point by the International Transport Forum^20^. This report estimated that “the share of the total population accessible to an average inhabitant within 30 minutes of driving is, on average, 50% for European metropolitan areas with 1 million inhabitants. For those with 6 million inhabitants, it is just 15%.” Absolute accessibility is affected by the size and density of the FUA.

Transport performance is a relative measure of accessibility which expresses the number of opportunities reachable within a certain travel time – i.e. absolute accessibility – with respect to the number of opportunities available within a certain radius^20^. It is calculated using the following formula:

$$T{P}_{i\kappa \lambda }=\frac{{\sum }_{j=1\,}^{n}{P}_{j}{\delta }_{ij}}{{\sum }_{j=1\,}^{n}{P}_{j}{\rho }_{ij}}$$

where:

*P*_j_ the population of destination zone *j*;

${\delta }_{ij}$ a binary variable equal to 1 when travel time from zone *i* to zone *j* is smaller than the determined travel time limit *κ* (30 minutes in our case) and 0 otherwise;

${\rho }_{ij}$ a binary variable equal to 1 when distance from zone *i* to zone *j* is smaller than the determined distance limit *λ* (10 kilometres radius) and 0 otherwise;

*n* the number of destination zones to be taken into account in the calculation (all in the specific case).

In contrast to absolute accessibility that grows with the size and density of available opportunities within reach, transport performance has been developed to control for the number of nearby destinations. By increasing travel times, congestion reduces the number of accessible destinations and as a result transport performance drops.

The location indicator measures the average travel time from an origin to all destinations, weighted by the population of destinations. It is given by the following formula^17^:

$$L{I}_{i}=\frac{{\sum }_{j=1\,}^{n}{t}_{ij}{P}_{j}}{{\sum }_{j=1\,}^{n}{P}_{j}}$$

where:

*t*_*ij*_ the travel time from cell *i* to destination zone *j*;

*P*_*j*_ the population of destination zone *j*;

*n* the number of destination zones to be taken into account in the calculation (all in the specific case).

The indicator is expressed in time units. Congestion increases average travel times and as a result the location indicator.

Potential accessibility measures the sum of population accessible from the origin zone per unit of travel time. Potential accessibility is given by the following formula^17^:

$$P{A}_{i}={\sum }_{j=1}^{n}\frac{{P}_{j}}{{t}_{ij}^{{\rm{\alpha }}}}$$

where:

*t*_*ij*_ the travel time from cell *i* to destination zone *j*

*P*_*j*_ the population of destination zone *j*

*n* the number of destination zones to be taken into account in the calculation (all in the specific case)

α is a parameter to control the decay function

Travel time is raised to the power of α to control for the importance of the role of distance, or time, between origin and destination. Values larger than 1 (i.e. a higher decay function parameter) increase the importance of relations over short distances^17^. By increasing travel times, congestion has a negative impact on the indicator.

### A summary of accessibility indicators

Absolute accessibility depends on the pre-determined travel time threshold. This permits a direct physical interpretation (population reachable in 30 min) but the fixed travel time threshold makes the indicator dependent on population density and the population size of the city. Transport performance was developed^20^ to address this issue by dividing the population reachable within a determined travel time by the total population within a determined distance. Potential accessibility indicates the average population reachable per unit of driving time. Without a pre-determined time threshold, it depends on the size, form and population distribution of the FUA. Finally, the location indicator measures travel time weighted by the population of destinations.

**Fig. 1:**
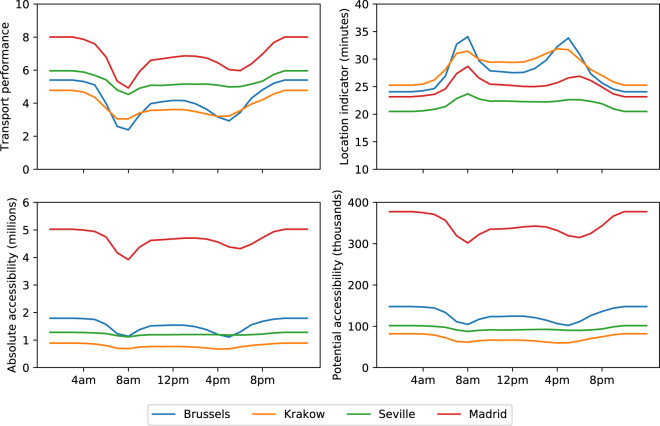
Hourly variation over the course of a day of the population-weighted average accessibility in Brussels, Krakow, Madrid and Seville according to the four indicators.

Accessibility at the level of FUA, city or commuting zone can be calculated as the population weighted average of accessibility according to the different indicators at grid level. The variation of the four indicators over the course of a weekday for the FUAs of Brussels, Madrid, Krakow and Seville is displayed in Fig. 1. The curves indicate the occurrence of morning traffic peak between 8:00 and 9:00 for the four cities but the afternoon peak appears to be smoother for Seville and Madrid. According to absolute accessibility, Madrid performs better than the other three cities in part because it is the largest one; it also has the best road transport performance, offering good access to nearby destinations. Seville performs worse than Brussels according to absolute accessibility, as it is smaller, but better according to transport performance, meaning that its road network offers better access to nearby opportunities. Accessibility in Krakow is the lowest – of all four cities – according to all measures indicating that Krakow is the city with lower access to fewer opportunities, also outside the peak hour.

Brussels and Madrid seem to be the cities most affected by congestion among the four as the drop of the accessibility indicators is sharper than in Seville or Krakow. During both the morning and afternoon peaks, transport performance of Brussels falls below that of Krakow and average travel time in Brussels (measured by the location indicator) increases above that of Krakow.

## Data Records

The accessibility dataset is available at the figshare repository^21^. For each populated 500 m by 500 m grid cell, it contains a value for each of the four accessibility indicators as estimated for free flow and morning-peak traffic conditions. The grid cells cover the FUA of 310 cities in EU27, the UK, Switzerland and Norway with population higher than 250 thousand people. The grid cells are represented by the coordinates of their centroid.

The dataset consists of 1,198,221 rows and 13 columns. The data attributes and their description are presented in Table 1.

**Table 1:** Data attributes.

Column name	Description
Lat	The latitude of the grid cell’s centroid
Lon	The longitude of the grid cell’s centroid
FUA_cd	The code of the FUA (city)
FUA_name	The name of the FUA (city)
Grid_POP	Population of the grid
AA_ff	Absolute Accessibility - free flow speed: Population accessible in 30 minutes
AA_ct	Absolute Accessibility - congestion from 8am to 9am: Population accessible in 30 minutes
TP_ff	Transport performance - free flow speed: Population accessible in 30 minutes over Population in radius of 10 km
TP_ct	Transport performance - congestion from 8am to 9am: Population accessible in 30 minutes over Population in radius of 10 km
PI_ff	Potential accessibility - free flow speed
PI_ct	Potential accessibility - congestion from 8am to 9am
LI_ff	Location indicator - free flow speed
LI_ct	Location indicator - congestion from 8am to 9am

**Fig. 2:**
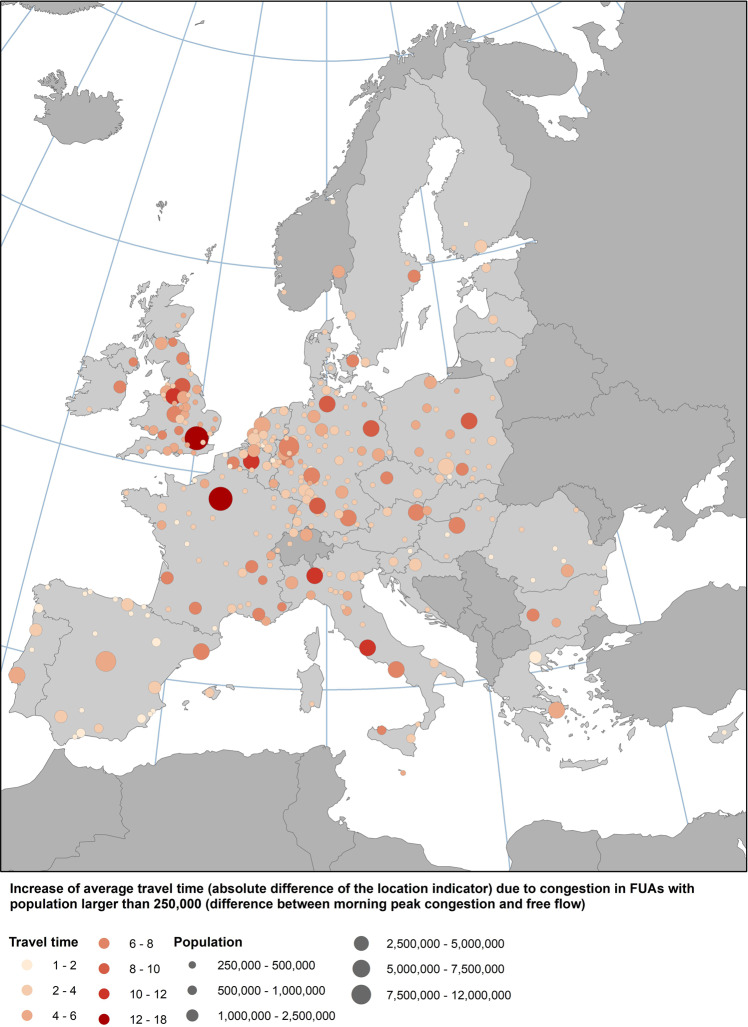
Impact of congestion on travel time. Results aggregated at FUA level for 310 European cities with more than 250,000 inhabitants.

The aggregate results of the impact of congestion on population weighed average travel time for all 310 cities are illustrated in Fig. 2. Congestion appears to have the highest impact on the location indicator in Paris, London, Brussels, Rome, Manchester and Milan.

**Fig. 3:**
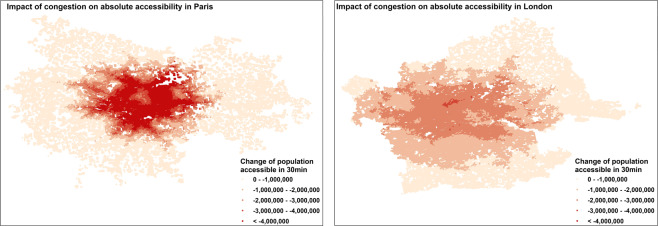
Impact of congestion on absolute accessibility in London and Paris at grid level. Absolute difference of population accessible from each grid in 30 min in the morning traffic peak (8am-9am) and in free flow conditions.

An illustrative example of the grid-level accessibility data is presented in Fig. 3 where the impacts of congestion on absolute accessibility at grid level in Paris and London are shown. The impacts in London, a polycentric metropolitan area, appear to be more spread across the FUA than in Paris where they are more concentrated around the centre.

## Technical Validation

We validated the data by comparing our travel time estimates with driving times from other data sources for a sample of origin-destination pairs drawn from all the cities. Our purpose has been twofold: validate the results of routing in similar traffic conditions and assess the potential of data to represent the impact of congestion.

The data source selected for validation is the Distance Matrix API of the Google Maps platform that provides estimates of driving times based on historic data. It allows determining the departure or arrival time of a trip as well as incorporating traffic information, in order to calculate travel times at different hours of the day and for different traffic conditions. Travel times are measured by routing on a road network, i.e. similarly to our chosen approach. Hence, the validation refers to the comparison of routing on two different networks (TomTom and Google) and, most importantly, the respective travel time information.

**Fig. 4:**
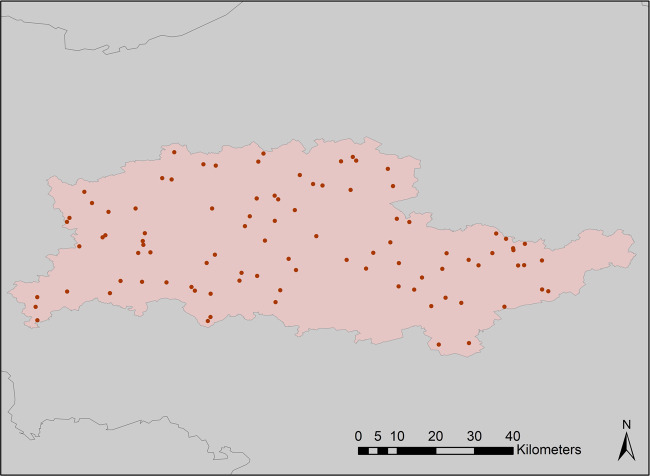
Randomly selected sample (n = 96) of grid cells in the FUA of Brussels used for the validation of the results. Travel times are calculated between pairs of these grid cells.

We measured travel times using Google Maps for a random sample of grid cells. The size of the sample varies with the size of the city; in larger cities such as Paris we measured journey times for almost 60 journeys while in smaller ones for less than 10 journeys. The sample of grid cells in the FUA of Brussels is illustrated in Fig. 4. In total, we calculated the travel times between more than 10 thousand origin-destination pairs.

**Fig. 5:**
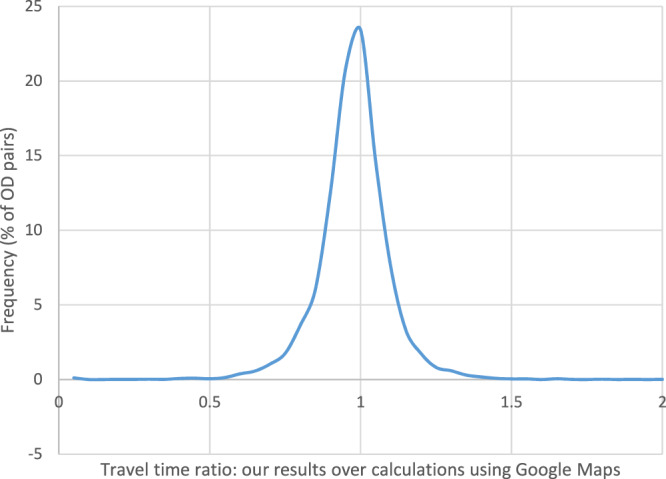
Comparison of our travel time calculations with measurements using Google Maps for 10,053 origin-destination pairs.

**Fig. 6:**
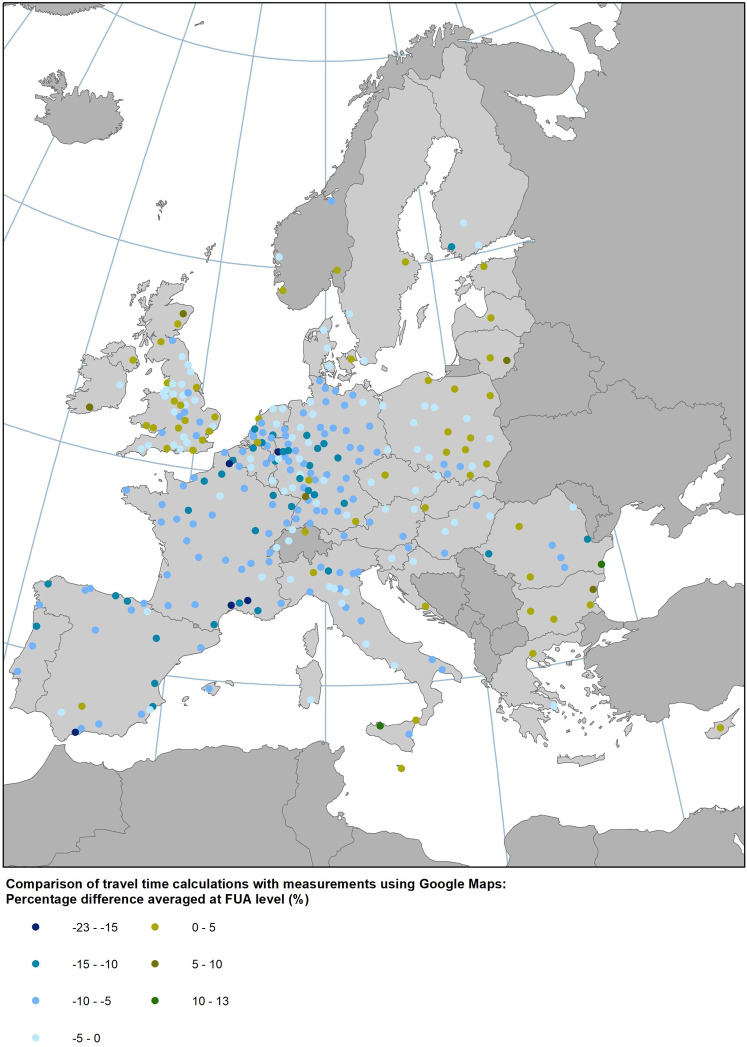
Percentage difference of travel time estimates with Google Maps at FUA level.

For the comparison with our travel time estimates, we measured travel times with Google Maps on a Tuesday, setting the departure time at 8:30 in the morning. We selected a Tuesday as it is the day of the typical week used for our calculations while the departure time in the morning peak was selected in order to compare our results corresponding to the 8–9 am time period (see Fig. 5). For almost 60% of the samples the percentage difference between the two was less than 5% and for almost 80% of the cases less than 10%. A linear correlation of the two travel times provides a good fit with an R^2^ of 0.94. In 70% of the samples, our travel time estimates are lower than those of Google Maps but for two thirds of them by not more than 10%. The majority of FUAs with a longer travel time based according to Google Maps are is Eastern and South-eastern Europe and the UK (Fig. 6).

**Fig. 7:**
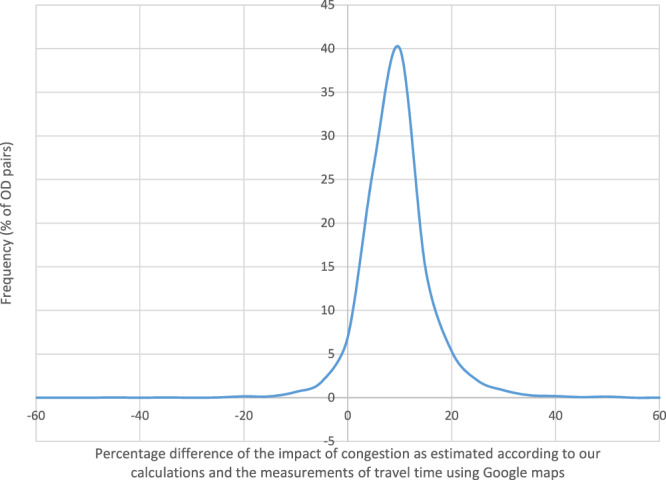
Comparison of the proportional impact of congestion as estimated according to our calculations and the measurements of travel time using Google maps for 3,146 origin-destination pairs.

For the comparison of the estimated impacts of congestion, we also measured travel time with Google setting the departure time at 2:00 in the morning when traffic is usually at its lowest. Then, we calculated the proportional change of travel time with (at 8:30) and without (at 2:00) congestion and compared it with the respective change calculated using our results, i.e. the proportional change of travel time at the 8–9 am period with respect to travel time in free flow conditions. The comparison of the estimated impacts of congestion was conducted for a sample of more than 3 thousand origin-destination grid cells. For 75% of the sample the difference between the two is less than 10% while the impact of congestion according to our calculations using the TomTom data seems to be on average 5 to 10% higher than that estimated based on the Google data (Fig. 7).

## Usage Notes

The accessibility data can be imported in any Geographic Information System, e.g. ArcGIS, MapInfo, QGIS etc., as well as other software or programming languages capable to process geographic coordinates such as Python, R, MATLAB etc. In ArcMap they can be added as a layer using the function that allows adding point data that contain geographic locations in the form of x, y coordinates.

Each data record contains a field with the name of the FUA that can be used to produce aggregate indicators for each functional urban area in Europe. Hence, it is possible to benchmark functional urban areas according to accessibility and analyse the impacts of congestion. Various comparisons can be produced, for example between FUAs of similar size, FUAs in the same country or region etc. Grid population is used to estimate population weighted averages and can provide an indication of population density.

The different accessibility indicators represent different aspects of accessibility. They have been calculated considering travel time from each grid cell to all other grid cells in the FUA. Free flow accessibility at grid level indicates the most and least accessible areas in each FUA, while accessibility with congestion can be used to identify the areas most affected by traffic. Grid level information offer great analytical opportunities as grids can form clusters based on user-defined characteristics.

Combined with other data such as travel demand, road network or public transport this dataset can be used to analyse solutions for the improvement of urban transport systems. Combined with fine resolution socio-economic data or information on deprivation it can help to analyse inequality issues. It can be also applied to study the relationship between access to opportunities and property prices. Finally, it can be combined with other files with spatial data on geographic boundaries to produce population-weighted averages of smaller spatial units such as municipalities.

## Data Availability

The Python scripts for the generation of accessibility indicators are available upon request.
